# The Life of Sir Humphry Davy

**Published:** 1831-04-01

**Authors:** 


### THE LIFE OF SIR HUMPHRY DAVY, Bart. By *Dr. Paris*, Quarto, 1831.

THE desire to become acquainted with the habits and peculiarities—in short, with the private life of all those who have attracted public attention by their genius or their discoveries, is almost universal—and hence it is that biography is a species of literature that is eagerly sought after, and greedily devoured. Sir Humphry Davy has excited as much sensation in the scientific world as Lord Byron did in the poetical;—and as Dr. Paris is fully as well calculated to portray the life of the one, as Mr. Moore is that of the other, the work at the head of this article will have a wide circulation, and will gratify the curiosity of a large circle of readers. It is hardly to be expected, however, that the perusal of this splendid volume will afford unmixed and universal delight. No man ever rose to the pinnacle of fame, even in the most useful sciences, without drawing upon himself the envy, or even the hatred of some of his contemporaries. The enemies of the illustrious dead will not relish the praise which may be justly accorded to their departed rival; while the ardent friends or idolators of the deceased, will be displeased with the slightest censure, or even with "faint praise."

The biographer, therefore, of an illustrious character so recently recalled from the stage of life, has a difficult task to fulfil, and can scarcely hope to please all parties. Honesty in this, as in every thing else, is the best policy; and we have no doubt that Dr. Paris laboured with equal zeal and candour, in the construction of the present biography:—we may add, too, with unexampled industry, considering that the compilation of the work was stolen from the hours of accustomed repose, and after the daily toils of a laborious profession were over.

As the costly quarto commemorating the life and discoveries of Sir Humphry Davy cannot circulate through the minute ramifications of our poor profession, we shall endeavour to give such a connected, but condensed view of the former (life) as may gratify, though not entirely satisfy curiosity; reserving for one or two separate articles, some account of the valuable scientific information with which Dr. Paris has enriched the biography of his friend.

Sir H. Davy was born in Penzance on the 17th December, 1778, of parents who could not boast of any very ancient or distinguished pedigree. His father was originally a carver in wood; but having come into the possession of some little patrimony, he followed this avocation rather as an amusement than as a source of emolument. He was not remarkable for any superior powers of intellect. The son, whose life we are commemorating, being placed at a preparatory seminary, made rapid progress, and excited the astonishment of the master. He was remarkable for the rapidity with which he could skim over a book, and yet be able to give a very good account of it; such was the quickness of his perception and the tenacity of his memory. The "Pilgrim's Progress" was the work which attracted most the attention of the young philosopher. He next studied history, and became a great lover of the marvellous, amusing himself and his school-fellows with the composition as well as the recital of tales of wonder. He became early fond of fishing and shooting, as well as some chemical experiments, such as the composition of detonating powder, &c. He went to Truro, in 1793, to finish his education, and here he evinced quickness and industry in his school exercises. The next year witnessed the death of his father, and Master Humphry was put apprentice to Mr. Borlasse, a surgeon-apothecary, his mother being engaged in the business of a milliner. In this new situation he still pursued his favourite pursuits—poetry, painting, philosophy—any thing rather than the compounding of physick. During his apprenticeship he attended a French emigrant priest to take lessons in that language; but never could acquire any thing like a good pronunciation of the French tongue. For the surgical part of the profession, he always evinced a decided distaste; but soon shewed his propensity for chemistry, often amusing himself with blowing up his master's bottles, as well as the rocks in his neighbourhood. Like Demosthenes, he had a defective utterance; and, like that orator, declaimed to the winds and waves on the rugged shore of his native coast. He had a remarkably bad ear, and never could learn even the tune of "God Save the King." He became a militia-man, at an early period, but never could emerge from the awkward squad, so backward was he in the acquisition of military tactics. Nevertheless he was not defective in courage or decision. Being bitten by a dog that was supposed to be mad, he took out his pen-knife, and cut away the part bitten without hesitation.

It was, no doubt, the nature and the scenery of the country around him, which directed his attention to chemistry and geology—since no one, now-a-days, believes that any "ruling passion" is ever an innate or irresistible propensity, antecedent to reason and observation. The prevailing bias of great minds may often be traced to some accidental, or even trivial circumstance, in early life. At the age of 12 years, Davy had finished an epic poem, entitled the Tydidead, celebrating the adventures of Diomede on his return from the Trojan war. No trace of this remains. Poetry and Love are nearly allied; and, at the age of 17, we find Davy desperately in love with a French lady at Penzance, to whom he addressed numerous love-ditties, none of which have descended to posterity. It was in early life that our philosopher and poet contributed to the "Annual Anthology," a work published at Bristol, in 1799, several compositions, which are reprinted in the present volume. They shew that the "bright and rosy hues of fancy," as Dr. Paris expresses it, were early cherished by Mr. Davy, and shone out even at the close of life, as we have seen in his "Consolations of Travel," already noticed in this Journal.

But we must pass over these juvenile effusions of his muse, to things of greater utility. The first chemical experiment of our hero, appears to have been performed for the purpose of discovering the quality of the air contained in the bladders of sea-weed. Mr. Davy's apparatus was very confined, as well as very clumsy; but a French vessel being cast ashore near Penzance, a glyster syringe escaped the waves, and was soon converted by the young chemist, for so we may now call him, into an air-pump, and even a pneumatic machine! This poverty of materials at the beginning conduced very much, in all probability, to Davy's fame in after life. Necessity is the mother of invention, and the great whet-stone of the human intellect. The subject of heat attracted much of the young philosopher's attention, and he devised a very ingenious apparatus, all things considered, for disproving the doctrine of Black, as to the materiality of caloric.

It was about this time that he was introduced to Mr. Giddy, now Mr. Gilbert—a circumstance on which much of his future prospects hinged. Mr. G. gave him the use of his library—introduced him to Mr. Edwards, a chemical philosopher, whose laboratory nearly threw young Davy into ecstatics. The late Mr. Gregory Watt, having gone to Penzance for his health, lodged in the house of Davy's mother, and here an intimacy commenced between the two, which proved another important link in the chain of the philosopher's life.

The geological warfare between the Neptunists and Plutonists, raged fiercely at this time, and Beddoes was a distinguished general in the Plutonic ranks. He wanted an assistant in his "Pneumatic Institution," at Bristol, and, in October 1798, Davy was the successful candidate. But in closing with Dr. Beddoes Mr. D. did not intend to give up the medical profession. Of his studies and experiments with the gases, the acids, and the metals, at Bristol, we cannot give any detail. It is evident that here the sphere of his chemical researches, and the ardour for chemical experiment were rapidly and greatly widened.

In the year 1801, through the negociation of Count Rumford with

Mr. Underwood, Mr. Davy was engaged in the Royal Institution of London, which was destined to prove the great theatre as well as the instrument of his future fame. He commenced as assistant-lecturer in chemistry, director of the laboratory, and assistant-editor of the journals of the Institution. He was allowed a salary of 100 guineas per annum, with a room, coals, and candles. He took possession of his new birth on the 11th of March, 1801. The first impression produced on Count Rumford by Davy's appearance, was extremely unfavourable, and the Count expressed to Mr. Underwood his great regret at having been an agent in the young philosopher's appointment. Davy's first lecture, however, removed all prejudice, and at its conclusion the Count expressed the highest degree of satisfaction.

Davy's uncouth appearance and address subjected him to many other mortifications, on his first residence in Modern Babylon. Johnson, the publisher, was then in the habit of giving weekly dinners to the choice spirits of the age, and Davy was invited to one of them. The host, however, thought it necessary to apologize to his polished guests, for the introduction of a visitor whose person was so forbidding, and whose pretensions were so humble. Fuseli was present, and highly energetic upon various passages of beauty in the poets; when Davy unfortunately observed that there were passages in Milton which he could never understand. "Very likely," said Fuseli, "but I am sure that is not Milton's fault."

In April of the above year Davy was elected one of the members of a society called the "TEPIDARIAN SOCIETY," consisting of 25 of the most violent Republicans of the day. Their meetings were held at the OLD SLAUGHTER'S, in St. Martin's Lane, and nothing but tea was allowed. Through the influence of this society Davy acquired considerable popularity. Meanwhile he performed his duties at the Royal Institution to the satisfaction of the managers, who soon appointed him lecturer, instead of *assistant*-lecturer. It appears that at this early period, Davy evinced that strong gastronomic propensity, which, in after years, displayed itself in a wider range of operations. At this time, also, he betrayed a tincture of *spinonism*, and he composed a poem under this name, since published with alterations and emendations by Miss Johanna Baillie. It is here re-published, but we pass it over. It was not till January 1802, that Davy's splendid career commenced. He then delivered a lecture to a large and enlightened audience, containing a masterly view of the benefits to be derived from the various branches of science. He represented the chemist as the ruler of all the elements that surround us, and which he employs either for the satisfaction of his wants or the gratification of his wishes. He describes the chemist as penetrating into the bowels of the earth, and searching the depths of the ocean, for the purpose of allaying the restlessness of his desires, or of extending the boundaries of his power. This lecture produced a great sensation in the audience, and it was printed at their request. He was now courted by eminent characters, and the manners and habits of the young philosopher soon underwent a considerable change. The fact is—Davy was not perfection! His vanity was excited, and his ambition raised by such extraordinary demonstrations of applause. The bloom of his simplicity was tarnished by the breath of flattery—and throwing off the native frankness, which constituted the great charm of his character, he assumed the garb and airs of a man of fashion! It need hardly be wondered at, if this new and inappropriate robe did not always fall in graceful draperies! Still the charms of the saloon never allured him from the pursuits of the laboratory. The crowds that daily attended his lectures were always gratified by newly-devised and highly illustrative experiments, conducted with great address, and explained in eloquent language.

There were some malecontent, however, or perhaps jealous spectators of this scene of success. His style was represented as too florid and imaginative, filled with inappropriate imagery, where violent conceits often usurped the place of philosophical definitions. This was Bœotian criticism; and the attic spirits selected other points of attack. They rallied him on the ground of affectation and mawkish sensibility. Dr. Paris has properly defended the philosopher from this charge, on the score of the mixed, and often unscientific audience to which the lectures were addressed. In popular lectures, the *manner* is often of greater power in attracting attention than the *matter*, and the decorations of language may well we pardoned, where the ultimate effect is beneficial. The same argument, indeed, might be fairly urged in published compositions. Many an excellent and useful work is neglected by the slovenliness and uncouthness of the language. Mr. Davy's audience were composed of the gay and the idle, who could only be tempted to admit instruction by the prospect of receiving pleasure. "They were children, who could only be tempted to swallow the salutary draught by the honey around the rim of the cup." The late Dr. Young, whose profound knowledge will not be questioned, soon lost his audience by the dryness of his style, and was dismissed from the Institution, lest it should become entirely deserted. In the laboratory, while carrying on his operations, he was as slovenly and desultory as the most rigid censor of his florid style in the lecture-room could desire.

From the exhaustion of metropolitan drudgery and scientific research, we find Mr. Davy in the year 1802, making an autumnal excursion through Wales. In letters now published we observe the enthusiasm with which the philosopher enjoyed and described the beauties and the grandeur of Nature. From the summit of Arran Benlynn, a mountain only inferior to Snowdon and Cader Idris, the philosopher beheld a scene of sublimity that excited his romantic imagination to the utmost stretch, and, no doubt, laid the foundation for excursions of wider range, and among Alpine and Apennine scenes of still greater majesty than Cambria can boast.

Returning to Modern Babylon, we are informed by his biographer, that Davy's life flowed on like a pure stream—not a gust ruffled its surface—not a cloud obscured its brightness. "In the morning he was the sage interpreter of Nature's laws; in the evening he sparkled in the galaxy of fashion." With wonderful facility he could accommodate himself to these opposite conditions of human life, readily casting aside the cares of study, and entering into the frivolous amusements of society. In closing the door of his laboratory, Davy opened the temple of pleasure—playing at billiards, frequenting the theatre, or reading the last new novel. "In ordinary cases, (says his eloquent biographer,) the genius of evening dissipation is an arrant Penelope; but Davy, on returning to his morning labours, never found that the thread had been unspun during the interruption." We do not quite coincide with Dr. Paris on this point. We believe the most intense studies are rather forwarded than retarded by hours of amusement or even of dissipation. If the strings of a harp are kept too long or too tightly braced up they will crack—and so it is with the human intellect:—if intervals of relaxation are not indulged, it will be soon worn out, or rendered unequal to vigorous exertion.

We must here pass over two chapters in Dr. Paris's work and Mr. Davy's life, chiefly occupied with the history of the philosopher's discoveries in chemistry at the Royal Institution, and come to the seventh chapter, when we find Mr. Davy gaining the prize offered by Napoleon for the best essay on the galvanic fluid, or rather of electric researches. We all remember the bitter animosity which France and England mutually entertained towards each other in the year 1807; and we cannot but be gratified and astonished when we find that, in spite of national prejudice and national vanity, the Bakerian Lecture of Mr. Davy made such an impression on the savans of Paris, that it was crowned with the prize of the First Consul! "Thus did the voltaic battery, in the hands of the English chemist, achieve what all the artillery of Britain could never have produced—*a spontaneous and willing homage to British superiority!*" This award was highly creditable to the French nation, and shews that—"the common-wealth of science is of no party, and of no nation—that it is a pure REPUBLIC, and always at peace."

The Bakerian Lecture for 1807, was attended by a crowded audience, and it would appear that the lecturer was kept so long in a state of excitement, that exhaustion and fever ensued, ending in a serious illness that caused an awful pause in his researches, broke the thread of his pursuits, and turned his thoughts into new channels of reflection. He always laboured under an impression that this fever resulted from contagion, to which he was exposed in one of the jails while making some experiments on fumigation. His medical attendants did not think there was any foundation for this opinion. The intellectual exertions of the philosopher were of the most intense kind at this time, and his mode of living by no means regular—perhaps not the most temperate. The greatest of all his wants was TIME—for he would not, like many men of science, give up the pleasures of the table. He was therefore obliged to work with redoubled ardour to make up for the time spent in company.

Having recovered from a long and severe illness (brain fever) Mr. Davy is presented to us in his character of an angler, with his green suit, many-pocketted coat, fishing tackle of various kinds, and coal-heaver's hat, &c. His shooting attire was equally whimsical, but he entertained a great and unnecessary dread of becoming game for some neighbour's gun, and therefore dressed himself in attire that would be sure to be seen and recognized at any distance. With all his science Mr. Davy does not appear to have been more successful in angling and shooting than other people.

In the year 1808 we find him delivering the Bakerian Lecture—and then prosecuting various chemical researches. In 1811, he delivers lectures in Dublin, and in the same year we find him directing his attention to the ventilation of the House of Lords. This undertaking proved a complete failure, which was a source of great mortification to the philosopher and pleasure to the wits of the day. Nevertheless the scientific renown of the philosopher had reached the ears of royalty, and the Prince Regent conferred on Mr. Davy the honour of Knighthood, in April, 1812. On the next day he delivered his farewell lecture at the Royal Institution, being now about to assume a new station in society. Other views of ambition than those of science opened upon his mind—the wealth he was about to command was to elevate him in the scale of society—his feelings became more aristocratic—he discovered charms in rank and titles which had before escaped his notice—and he no longer viewed patrician distinctions with philosophic indifference. How far this revolution contributed, or was likely to contribute to an increase of his happiness, we shall not say; though he must be void of all reflection who can fail to form an opinion on the subject. In April (an ominous month) 1812, Sir Humphry married Mrs. Apreece, the widow of S. A. Apreece, Esq. the daughter and heiress of Charles Kerr, Esq. of Kelso, and possessing a very considerable fortune. The happy pair made a tour through Scotland, and were every where greeted with the most flattering attentions.

Some time previously Sir Humphry had entered into a kind of partnership with Mr. Children and Mr. Burton in a gunpowder manufactory, and the correspondence between these partners or company occupies some space in the volume before us, but these we pass over. The ninth chapter of the volume is taken up with various chemical discoveries of the philosopher, and with his work on agricultural chemistry. These we also pass by untouched. In the year 1813, Mr. Faraday introduced himself to Sir Humphry, having taken a great liking to chemical science in preference to the business of bookselling, to which he was apprenticed. Sir H. appointed him as assistant in the laboratory, and in October, 1813, took Mr. F. with him abroad, as his assistant and amanuensis.

Napoleon having granted a passport to Sir Humphry and suite to go to France, and ultimately to Italy, the party landed at Morlaix, and were instantly arrested by the authorities of the place, who could not believe that their passports were genuine. They were soon set at liberty, however, and proceeded to Paris, where Sir H. found his old friend Mr. Underwood, one of the detenu's. Mr. U. conducted Sir Humphry to the Louvre, through the galleries of which the philosopher paced, without ever directing his attention to a single painting on either side! The only exclamation he uttered was—"what an extraordinary collection of fine frames"! Mr. U. forced his attention to Raphael's picture of the transfiguration. Davy's reply was still laconic—"I am glad I have seen it," and passed on. The statues in the lower apartments of the Louvre excited no remark or attention from the philosopher! "while the marble glowed with more than human passion, the living man was colder than stone." He betrayed the most complete apathy and indifference when he was shewn the Belvidere Apollo, the Medicean Venus, the Laocoon; but the sight of Antinous, treated in the Egyptian style and sculptured in alabaster, brought forth a most enthusiastic ejaculation of vivid surprise. "Gracious powers, what a beautiful stalactyte!" Here we have a philosopher who, with the glowing fancy of a poet, was insensible to the divine beauties of the sister arts. "Let the metaphysician," says Dr. P. "unravel the mystery if he can—the biographer has only to observe that the Muses could never have danced in chorus at his birth." We suspect there was affectation at the bottom of all this. The statues of antiquity always excite vivid emotions in every mind at all imbued with classical literature, however deficient in taste for sculpture, painting, or poetry. It is scarcely possible therefore that the Apollo and the Venus de Medicis could have failed to call forth emotions in Sir Humphry's breast, had he not determined to suppress them. On the following day he visited the colossal elephant, intended to form a part of the fountain then erecting on the site of the Bastile, and with this he appeared to be highly delighted.

The savans of Paris hastened to pay their respects to the great English philosopher. He attended the Institute, and was placed on the right hand of the president. While there, his lady met with a curious adventure, illustrating the state of the police under the great Napoleon. She had walked into the Thuilleries garden with her maid, dressed in a small hat then worn in London, while the Parisian belles wore huge head-dresses, such as now obstruct our view in all public places. The Parisians were lost in amazement at the sight of the small bonnet, and assembled in crowds around the strange exotic. In consequence of this a police inspector warned her ladyship off, lest she should cause a "*rassemblement*," to the danger of the state! The assemblage, however, rapidly became so great that it was necessary to send for a corporal's guard, and conduct the small bonnet out of the gardens with fixed bayonets! The philosopher appears to have soon lost his popularity in Paris, principally perhaps from his own awkwardness, prejudices, not to say pride. He seems also to have incurred the displeasure of the Emperor, and we find him on his way to Lyons in December, 1813. He remained a month at Montpellier, where he employed himself in experiments on iodine and various fuci and ulvæ of the Mediterranean. In February, 1814, he proceeded to Nice—crossed the Col di Tende to Turin, and thence reached Genoa, the new and magnificent road from Nice to Genoa not then being open. From Genoa he proceeded to Florence—and from Florence to Rome, where he still pursued his chemical investigations. Naples attracted the philosopher's attention, on account of Vesuvius, the grand laboratory of Nature. Returning thence he crossed the Simplon to Geneva, where he pursued his studies for a time, and then visited the Tyrol, Venice, &c. Our philosopher returned to England by the Rhine, in 1815, and directed his attention to the *fire-damp* of our great collieries, where a dreadful explosion had then excited the sympathy of all humane persons in England. The coal appears to part with a portion of carburetted hydrogen, when newly exposed to the air. On some occasions the pit-men have opened with their picks crevices or fissures, in the coal or strale, which have emitted as many as seven hundred hogsheads of *fire-damp* in a minute. These *blowers*, as they are called, have been known to continue in a state of activity for many months, or even years together—shewing the tremendous pressure under which the gas must have lain! On the approach of a candle the gas is instantly kindled—the expanded fluid drives before it a roaring whirl-wind of flaming air, which tears up every thing in its progress—scorching some of the miners to a cinder, and burying others under enormous ruins shaken from the roof. When thundering to the shafts, it converts the mine, as it were, into a gigantic piece of artillery, and wastes its fury in a discharge of thick clouds of coal-dust, stones, and timber, together with the limbs and mangled bodies of men and horses! All the stoppings and trap-doors of the mines being blown down by the violence of the concussion, and the atmospheric current being entirely excluded from the workings, those miners who survive the discharge are doomed to the more painful and lingering death of suffocation by the AFTER-DAMP, as it is termed, which immediately results from the combustion, and occupies the vacuum necessarily produced by it. Here Dr. Paris introduces the relation of one of these horrible catastrophe's, as drawn up by the Rev. I. Hodgson, and which is prefixed to the funeral sermon preached on that melancholy occasion. This we must pass over. It is well calculated to harrow up the feelings, and judiciously—perhaps artfully, brought forward to enhance the value of the safety-lamp, which is next introduced to the reader's notice. We need not dwell on the mechanism and rationale of the SAFETY-LAMP, both of which are well known to all our readers. We agree with the eloquent biographer, that the invention of this lamp was one of the most splendid of Davy's triumphs. He not only disarmed fire-damp of its terrors—but enlisted it into his service in consuming itself. "He constructed a lamp, which has been truly declared to be as marvellous in its operation, as the storied lamp of Aladdin, realizing its fabled powers of conducting in safety, through fiends of combustion to the hidden treasures of the earth. We behold a power which, in its effects, seemed to emulate the violence of the volcano and the earthquake, at once restrained by an almost invisible and impalpable barrier of net-work—we behold as it were, the demon of fire taken captive by science, and ministering to the convenience of the miner, while harmlessly fluttering in an iron cage." The success of the wire-gauze lamp is now complete. It has been used for many years with entire security.

The biographer now proceeds to exhibit our philosopher in a new scene of action or field of enquiry—investigating amid the ruins of Herculaneum, the nature and effects of the volcanic eruption which overwhelmed that city in the reign of Titus—and endeavouring to unfold the mouldering archives which had been recovered from its excavations. In his way to Italy, by the Rhine and the Danube, he made numerous experiments on the formation of fogs and mists over rivers and lakes. The results of his observations are printed in the Philosophical Transactions. These results establish the law that the formation of mist on a river or lake, never takes place, if the temperature of the water be lower than that of the atmosphere—not even though the latter should be saturated with vapour. The unrolling of the Herculanean M.S. did not go on well, and was discontinued at the end of two months, when Sir Humphry returned to England, and was soon after elected President of the Royal Society, viz: on the 30th Nov. 1820. Dr. Paris discusses the question, whether a President of this learned Society should be a man of general acquaintance with all departments of science, but attached particularly to no one—or whether he should be a philosopher actively engaged in the pursuit of a particular branch of knowledge? He is inclined to favour the former, and we confess we lean to the same side. "It is not in human nature to believe that the looker-on, and he who plays the game, are alike indifferent to the cards." An influential personage in the Royal Society shrewdly remarked to Dr. Paris, on a late occasion, that they did not require an Achilles to fight their battles, but an Agamemnon to command their forces.

Be this as it may, one thing is certain—that Sir Humphry Davy retired from office under the frown of a considerable party. "As a philosopher, his claims to admiration and respect were allowed in all their latitude; but when he sought for the homage due to patrician distinction, they were denied with indignation. How strange it is, that those whom Nature has placed above their fellow men by the godlike gift of genius, should seek from their inferiors those distinctions which are generally the rewards of Fortune." Nothing can be more clear than that Sir Humphry's weak point was vanity, which shewed itself in this pitiful hankering after patrician honours and distinctions. "We can only lament (says his biographer) the weakness from which the choicest spirits of our Nature are not exempt." Davy was no solitary instance. Congreve, in his interview with Voltaire, prided himself upon his fashion rather than upon his wit—Byron was more vain of his heraldry than of his Childe Harold—and Davy sighed for patrician distinction in the chair of Newton! Well may Dr. Paris ask—"Will philosophers never feel, with Walpole, that a genius transmits more honour by blood than he can receive?"

Dr. Paris next gives a sketch of the elaborate experimental enquiry of Sir Humphry, into the chemical nature and causes of the well-known corrosive action of sea-water upon metallic copper, with the view of obviating that serious evil in naval economy. "Paradoxical as it may appear, (says Dr. P.) the truth of his theory was completely established by the failure of his remedy!" As we shall have occasion to recur to this subject in a separate article, we shall say no more here. In 1824 we find the philosopher visiting Heligoland, Norway, Sweden, and other places, in prosecution of the above researches; but the failure of his plan evidently produced in his mind a degree of chagrin and disappointment wholly inconsistent with the merits of the question. At this time, the philosopher's bodily health was unequivocally declining, and he began to think of secession from a public station, with the hope of finding in retirement and change of air, a restoration of that blessing, health, without which honours are of little avail! In the year 1826 he complained of debility, and that he could not hunt and shoot with the same energy as twenty years previously. On returning to London he complained to Dr. Paris of palpitation of the heart, and of an affection of the trachea, which excited fears in his mind. Towards the close of the same year, his indisposition increased—his powers were becoming undermined—his spirits oppressed—and his energies subdued. On his way from the country to town, he was seized with an apoplectic attack, from which he was rescued by prompt and copious depletion, but not without a remaining paralysis. He was then advised change of air; and the southern part of Europe (for what reason, medicinally speaking, we know not) was selected for the restoration of health. He therefore departed for Italy. In March 1827, we find him at Ravenna, a spot which he had chosen, as "one of solitude and repose," being out of the way of travellers, and where the monuments of ancient power are not without interest. He was accompanied by his brother, Dr. Davy, who then considered Sir Humphry as a convalescent, though he had travelled from England, and passed the Alps in the depth of Winter—a procedure which probably conduced more to his partial recovery than the air of Ravenna, situated in the midst of marshes. In a letter from Ravenna, he says, "I ride in the pine forest, which is the most magnificent in Europe. Imagine a circle of 20 miles of these great fan-shaped pines, green sunny lawns, and little knolls of underwood, with large junipers of the Adriatic in front, and the Apennines still covered with snow behind. The pine-wood partly covers the spot where the Roman fleet once rode—such is the change of time!" He now resigned the chair of the Royal Society, and Mr. D. Gilbert was elected pro tempore.

By a letter dated July 1st, at Salzberg, we find him desponding. The confident expectations of recovery indulged by his physicians had not been realized. The tendency to congestion in the head could only be kept at bay by the most rigid abstinence, vegetable diet, and frequent bleedings. Still he entertained hopes of final recovery. In October 1827, we find him again in England, where he consulted the most celebrated writers on the nervous system, who all "had a good opinion of his case." In November he writes from Firle, near Lewes. He is very weak; but still occupying two or three hours daily in the construction of his Salmonia, a long review of which is here given by his biographer. He proceeded to Italy; and the last letter he ever wrote, is dated Rome, February 6th, 1829. He tells his friend Mr. Poole that he was "wearing away his time" in the Eternal City, "a ruin among ruins." He says that he sometimes thinks of the lines of Waller, (we believe it was Pope,) and seems to feel their truth.

> "The soul's dark cottage batter'd and decay'd,
> Lets in new lights through chinks which time has made."

His eloquent biographer here enters into a very long analysis of the philosopher's last work, "the Consolations of Travel," of which we gave an account in a late number. This analysis occupies 27 quarto pages, and, considering the smallness of the original volume, is, we think, inordinately stretched out—more especially as it must have been read by every one who peruses the biography, and by many thousands of others. We agree with Dr. Paris in the following sentiment. "In the extracts which have been introduced from this his last work, I trust the pledge that was given in the earlier part of these memoirs, has been redeemed, by shewing that a powerful imagination is not necessarily incompatible with a sound judgment—that the flowers of fancy are not always blighted by the cold realities of science—but that the poet and the philosopher may, under the auspices of a happy genius, mutually assist each other in expounding the mysteries of Nature."

The history of our philosopher draws to a close. He had been resident some months at Rome, where he occupied the second floor of a house in the *Via di Pietri*, leading out of the Corso—a house which we often paused to contemplate a few months after the philosopher left it—and where he had another paralytic seizure. Lady Davy, on this occasion, made a most rapid journey from England to Rome, namely, in 12 or 13 days—a rapidity which was often the theme of wonder in the Eternal City afterwards. With that restlessness which characterises the complaint under which the philosopher laboured, he became extremely anxious to quit Rome, and to get to Geneva—a desire which does not surprise us, considering the depressing atmosphere of the city of the Cæsars, and the elastic breezes of Switzerland! His friends were naturally anxious to gratify his wishes, and Lady Davy preceded him on the journey, in order to prepare comfortable apartments for her dying husband. He arrived at the COURONNE, in Geneva, on the 28th of May, accompanied by Dr. Davy, Mr. Tobin, and his servant. He made a hearty dinner, and admired the fish—was very cheerful—and joked with the waiter on the cookery. He went to bed at 11 o'clock, and his servant, who slept near him, was called to him in the night, when he summoned Dr. Davy, who found him in articulo mortis. He died at 3 o'clock in the morning. He was buried respectfully in the cemetery of PLAM-PALAIS, where we sighed over his grave four months after his decease. A tablet in Westminster Abbey, placed there by his widow, is the only public memorial, yet erected to the memory of this distinguished philosopher. We shall conclude this rapid glance of Dr. Paris's biography in the words of its author.

"The fame of such a philosopher as Davy can never be exalted by any frai memorial which man raise. His monument is in the great temple of Nature. His chroniclers are Time and the Elements. The destructive agents which reduce to dust the storied urn, the marble statue, and the towering pyramid, were the ministers of his power, and their work of decomposition is a perpetual memorial of his intelligence."

The biographer had a difficult task to perform. Davy's life was so much connected with the history of his chemical discoveries that it was necessary to keep the one in close parallel with the other. The life of a philosopher, indeed, seldom affords materials enough, independent of his works, for the biographer. Dr. Paris has executed his task with his usual ability—and with great apparent impartiality. While he has set forth the genius and talents of the philosopher, he has not entirely drawn a veil over his failings, as our readers will have perceived.

We have heard it remarked that Dr. Paris has swelled the volume unnecessarily by private letters, and by long extracts from the published works of Sir Humphry Davy. It is difficult to discriminate on such occasions. The private letters of a man often disclose his character more than formal publications. In respect to the extracts, those from "Consolations of Travel," or the last words of a philosopher, exhibit a nearer insight into the thoughts of the author than any of his scientific productions, and their insertion in a biography is more excusable.

